# Equine Milk Production and Valorization of Marginal Areas—A Review

**DOI:** 10.3390/ani10020353

**Published:** 2020-02-22

**Authors:** Nicoletta Miraglia, Elisabetta Salimei, Francesco Fantuz

**Affiliations:** 1 Dipartimento Agricoltura, Ambiente e Alimenti, Università degli Studi del Molise, Campobasso 86100, Italy; miraglia@unimol.it; 2 Scuola di Bioscienze e Medicina Veterinaria, Università degli Studi di Camerino, Camerino MC 62032, Italy; francesco.fantuz@unicam.it

**Keywords:** equine milk, dairy equine chain, dairy equine management and feeding, biodiversity, landscape, pasture

## Abstract

**Simple Summary:**

The revaluation of equine milk for human consumption is showing an increased interest from a scientific point of view. As practical relapse of the peculiar characteristics of horse and donkey milk, and their potentialities as food products, the dairy equine enterprise is developing worldwide. The milk production can therefore contribute to the whole equine industry, but crucial factors still need to be elucidated. Aiming to promote advances of knowledge on the dairy equine enterprise, aspects of management of the dairy horse and donkey are reviewed in the frame of marginal areas, with a special focus on dam and foal feeding, and welfare, besides milk quality.

**Abstract:**

The equine dairy chain is renewing the interest toward horse and donkey breeding for the production of milk with potential health promoting properties. The dairy equine chain for human consumption could contribute to the rural eco-sustainable development for the micro-economies of those areas threatened by marginalization. As a part of the whole equine industry, and its possible impact in the modern and future society, the main traits of the equine dairy enterprise are reviewed with a special focus on management of animals and milk. Equine milk compositional and nutritional peculiarities are described as also related to milk hygiene and health issues. Scientific and technical aspects of the feeding management are considered in the frame of the emerging dairy equine enterprise, where pasture is an essential element that allows to match production goals for horses and donkeys, biodiversity preservation, as well as landscape safeguard.

## 1. Introduction

Equine breeding represents one of the most promising activities in rural development, which is considered a key strategy for restructuring the agriculture sector by means of diversification and innovation [1]. The equine species are involved not only in activities concerning their use for work and tourism, but also in niche activities related to the production of food and non-food products [2,3]. The high versatility of the equine species represents a strong argument for the conservation of endangered equine breeds and populations [4,5]. Many breeds occupy special niches and contribute to the biodiversity due to their own genetic characteristics, coming from adaptive mechanisms developed in centuries of evolution in specific local environments [6,7]. Consequently, policies for the safeguard of endangered equine breeds and autochthonous populations have been developed, also considering the recovery of the relationship among humans, animals, and territory, as a ’system integrator’ of the rural eco-sustainable development [8,9]. The renewed interest toward equine milk and derivatives is today sustained by the emerging dairy equine enterprise, which is developing in France, Italy, Mongolia, China, Kazakhstan, Kirgizstan, Greece, Germany, and many other countries [10,11,12]. 

As a part of the whole equine industry and its potential impact in the modern and future society, the equine dairy enterprise is described in its main traits. Based on peculiarities of equine milk for human consumption, the essential features of management of animals and milk are reviewed in the context of marginal areas. Aspects of nutrition of the dairy equids are examined in the frame of those areas where pasture and natural meadows represent the main land use, as a further contribution to landscape safeguard.

## 2. Equine Milk: Properties, Potentials, and Benefits

The nutritional and therapeutic peculiarities of equine milk are known since ancient times, as Hippocrates [13] and Herodotus [14] described in the 5th century BC. Moreover, the consumption of koumiss (or airag), i.e., a traditional drink made in Central Asia, according to a nomads’ recipe [15], is reported in literature, not only as an ingredient of the traditional “white diet” of the Mongolian steppes population [16], but also as a popular remedy for a variety of diseases [17,18]. The traditional use of donkey milk is also reported in China and South America for the treatment of many illnesses [19].

Recent scientific findings on the equine milk compositional peculiarities and their potential health promoting properties have increased interest toward its use for human consumption, especially for sensitive consumers, such as children with allergies to cow’s milk protein, as well as immunocompromised or debilitated people [10,20]. In Europe, the dairy equine enterprise started up in France as part of a project on animal diversity preservation [21], and spread out in many marginal areas of the world where these monogastric herbivores are well adapted to difficult environments, with scarce availability of forages, often of poor-quality. Today, equine milk is mainly marketed for human consumption as raw, pasteurized, or freeze-dried [22], and as fermented derivatives [15]. In Italy, the price of donkey milk ranges from 9 to 15 €/L of raw milk, 14 to 17.5 €/L of pasteurized milk, and 27.5 to 36 €/100 g of powdered milk, either spray dried or lyophilized [23,24]. Equine milk is also used in the non-food sector, as an ingredient in cosmetic products [25]. Data on the worldwide production of equine milk are not available, but equine milk has been reported to be consumed by 30 million people [26]. It should also be considered that consumer cognizance of equine milk and derivatives is so far limited, as well as common awareness of its local availability [18,27]. Besides communication gaps to be overcome, the emerging niche market of equine milk raises questions on appropriate management strategies of dam and foals, as mainly related to animal nutrition as well as environmental issues, besides food security and animal welfare.

### 2.1. Equine Milk Compositional and Nutritional Features

Table 1 summarizes the average horse and donkey milk gross composition and energy content from the recent literature. Values from human and cow milk are given for comparison. It should be considered that equine milk components are mainly affected by nutrition, length of lactation, and health status of the mammary gland, besides genetics. Equine milk has a high water content and shows a lower fat content than human and cow milk (Table 1). The milk fat globules diameter, likely related to lipid digestibility, is reported to be lower than in human and bovine milk [28].

Horse and donkey milk are closer to human milk in terms of both protein and lactose content than cow milk (Table 1). On this regard, it is worth noting that, although about 50% of the world population is lactose intolerant, the daily intake of 14 g of lactose is usually well-tolerated [28]. Moreover, the content of lactose is lower in fermented milk. From a nutritional point of view, it is also important to highlight the hypocaloric content (Table 1) that makes equine milk an inadequate food for infants, when not supplemented with vegetal oil (about 40g L^−1^) [28].

The ash content of equine milk (Table 1), which is intermediate between human and cow milk, shows a decline throughout the lactation consistent with Ca and P concentrations in milk [15,20]. Although the absolute values of Ca and P in equine milk are reported variable, and in average higher than in human milk, the Ca:P ratio is reported to be in average 1.3 and 1.72, respectively, for donkey and horse milk, while it accounts for 1.7 and 1.23 in human and bovine milk, respectively [31,32,33]. As a further dietary consideration, the mineral content of milk is not reported to be influenced by the maternal diet in mammalians, except for Se and I [34,35].

Pieszka et al. [36] reviewed the level of fat-soluble vitamins (A, D, E) in mare milk and found them consistent with values reported for bovine milk. Donkey milk is reported to contain a higher level of vitamin D [37] but it displays very low contents of vitamins A and E, as probably related to the low-fat content [28]. Among the water-soluble vitamins, pyridoxine, pantothenic acid, cobalamin, and vitamin C have so far been detected at high levels only in mare milk [28,36].

After the first clinical evidences on the successful use of equine milk in children with multiple food allergies, reported in 1992 and 2000, respectively, for donkey and horse milk [38,39], donkey milk has mainly been the subject of numerous studies about its use in the diets of children affected by cow’s milk protein allergy, thanks to its high palatability, due to the high lactose content, and low allergenicity, related to the nitrogenous components [28,40]. Equine milk with high hygiene characteristics, and properly supplemented from a nutritional point of view, has been confirmed as a promising alternative in the dietary treatment of children affected not only by Immunoglobulin E-mediated cow’s milk protein allergy, but also by food protein-induced enterocolitis, occurring in the first six months of life [40,41]. However, findings on the efficacy of equine milk use in the fulfilment of nutrient requirements of children cannot be so far considered conclusive, as they need to be confirmed by larger studies [27]. For these reasons, the use of donkey milk is nowadays considered an ingredient in a solid-food diet, or after the first year of life for children [42].

In regards to the allergenicity of equine milk, the proteomic profile of equine milk has been extensively studied in recent years [43,44], and microheterogeneity is displayed due to genetic variants and post-translational modification [28]. A significant effect of the breed and stage of lactation on gene expression and milk composition, and the association among genetic polymorphisms, gene expression, and milk protein and fat contents have also been observed in mare milk [45,46,47]. This leads to the relevant role of the dairy equine enterprise in the survival of equine breeds, e.g., Lipizzan, Icelandic, German Warmblood, Akhal-Teke, Franches-Montagnes, Comtois, Italian Heavy Draught, Russian Heavy Draft, and Polish Coldblood among horses; and Poitou, Zamarano Leonés, Burro de Miranda, Ragusano, and Amiata among donkeys, and in the preservation of environment, landscape, and vegetal diversity of areas where they are adapted to live [2,24].

### 2.2. Functional and Bioactive Compounds

Milk, besides allergens, is a source of many bioactive and functional compounds, i.e. metabolites, enzymes, hormones, trophic, and protective factors that are involved in proper growth and nutrition in newborns [48], or in proper secretion of the mammary gland [49]. 

Among the bioactive and functional proteins detected in milk, there are enzymes active in protection against protozoa, bacteria, and viruses, e.g., lysozyme and lactoferrin [48]. Lysozyme accounts for 10.5% and 21% of whey proteins, respectively, in horse and donkey milk, but only 5.5% of whey protein in human milk; on the contrary, a higher level of lactoferrin (26.6% of whey protein) is detected. Lactoferrin in horse and donkey milk accounts, on average, for only 7% and 4.48% of whey protein, respectively [11,50,51]. Lysozyme activity was found unaffected by thermal treatment at 72 °C up to 3 min [52,53]. 

Other enzymes in milk are of technological relevance, such as alkaline phosphatase representing an index of pasteurization efficiency, with activity reported to be about 100 mU L^−1^ in thermal treated equine milk [22]. 

In mammalian milk, hormones and growth factors have also been detected and classified as bioactive peptides [54] derived from the maternal metabolism. Among them, there are leptin, insulin, ghrelin, Insulin-like growth factor 1 (IGF-1), and thyroid hormones that are involved in the central regulation of food intake and in the maintenance of energy balance. Their role in milk may be related to the regulation of growth, to the development and maturation of the neonatal gut, and of the immune and neuroendocrine system of the newborn [48]. Considering the species-specificity of many proteins, it is worth noting that leptin has been measured as human equivalent in both horse and donkey milk, and human-like ghrelin, IGF-1, and triiodothyronine (T3) were measured in donkey milk [15,20]. It is worth noting that the milk T3 content was affected by the diet in lactating donkeys [55]. The role of variations in the maternal hormone status of equids, as related to both physiological status, and how intensive husbandry strategies might interact with their adaptive capacities in the farming environment, deserves attention and needs to be further considered.

Bioactive peptides are also encrypted in the sequence of milk proteins and are released from them following enzymatic proteolysis, under gastrointestinal digestion or during fermentation. These dietary components exert health promoting, i.e., antimicrobial, antihypertensive, antioxidant, antithrombotic, immunomodulatory, antiproliferative, and opioid activities in the organism, beyond their nutritive value [56,57,58,59]. It should be noted, however, that technological treatments carried out to prolong milk shelf life could considerably affect structure, as well as the functional and nutritive properties of milk components, especially peptides and proteins, that might lead to a greater susceptibility to infection and/or the development of allergies [60]. 

In immunonutrition, the antioxidant properties of nutrients, such as alpha-tocopherol and beta-carotene are known, but increasing scientific evidence suggests the role of dietary lipids in the regulation of neonatal immune function and in the severity of symptoms of allergies [61]. Recent studies show the interesting free fatty acids profile of equine milk, with saturated fatty acids content (50%) lower than that reported for goat and sheep milk, and a higher proportion of monounsaturated fatty acids and polyunsaturated fatty acids (PUFA) than ruminant milk [28]. A balanced ratio between n3PUFA and n6PUFA, respectively considered anti-inflammatory and pro-inflammatory nutrients, is reported for horse and donkey milk [62]. Moreover, in regards to the variability of the lipid fraction, Martini et al. [63] observed an increased content of oleic, palmitoleic, and vaccenic acids considered with a positive effect on human health, and a lowered concentration of stearic acid in donkey milk samples collected in the winter.

The atherogenic and thrombogenic indices, calculated on fatty acid composition, candidate equine milk as an interesting food for people with allergic and inflammatory conditions [28,64]. Moreover, the fatty acids profile detected after in vitro digestion shows significant differences depending on the milk sources, with a prevalence of saturated fatty acids released from both human and donkey milk [65]. Heat damages have been observed in donkey’s milk on functional lipid compounds, which may also directly and indirectly influence gut environment and immunoinflammatory functions [66,67]. 

The recent advances of knowledge on the claimed nutraceutical properties, here summarized, suggest that, when scientifically demonstrated, the added value of the equine milk should be properly exploited in the dairy equine enterprise.

## 3. Dairy Equine Management and Nutrition

### 3.1. Equine Milk Yield and Management of the Dairy Equine Enterprise

The core of the dairy equine enterprise is related to the management of dams and foals, and of the milking practice, showing important differences from the conventional dairy species. Firstly, dams and foals live together until weaning, which occurs at 7 months (for foals) or later; dams won’t start to be milked before 20 d from foaling [10,68]. Secondly, since the equine mammary gland is characterized by small volume, and milk is mainly alveolar [69], milk harvesting can be carried out many times per day. In the Steppes of Central Asia, mares are milked 4–5 times per day [70], while in more intensive dairy farms located in Europe, mares and jennies are frequently milked depending on consumer demand, up to eight times a day [10,62,71]. Milking is carried out at least 2 hours after foal separation from the mother [70,72]. This distinctive trait of the dairy equine enterprise introduced the neologism “milking session”, i.e., the interval from foal separation up to the end of each milking [62]. It must be noted that milk ejection is not reported to be affected by the presence of the foal during milking in the dairy donkey farm [68], while it is recommended in the dairy horse farm for a complete oxytocin release [10]. In this regard, the selection for milkability of mares would greatly improve the milking routine, reducing the labor costs [73]. 

Milk harvested per milking session is reported to range within 500–2000 mL and 200–900 mL for mares and jennies, respectively [62,70,74,75,76,77], regardless of the milking technique used (mechanical or manual).

The available literature data on daily equine milk yield have been obtained under different methodological approaches, which partially explains the high variability of values reported in Table 2. The daily milk production is estimated to be 15–35 g kg^−1^ bodyweight [10,29,78,79]. However, literature data are inconclusive, as the value recently estimated for the dairy donkey, i.e., 12 g milk kg^−1^ body weight, shows [80]. Todini et al. [81] reported an average milk yield per milking of 2.68 mL kg^−1^ bodyweight.

Milk yield is affected by many factors, including the farming system, nutrition and feeding, strategy and type of milking (manual or mechanical), individual milkability, stage of lactation, and size and body condition of animals, besides genetics [11]. Because of the lack of standardized methodologies in equine milking studies, the effect of the breed on dairy performances of mares and jennies is not currently defined. According to Doreau and Martin Rosset [10], any breed can be milked, provided the animals accept the milking procedure. 

The farming system is a major cause of the observed variability in equine milk production, as reported for pastoralist areas of the Steppes of Central Asia [70,85], or for more intensive systems, described for both koumiss and dairy donkey farms. In the latter, shelters are available on pasture, and milking is usually carried out in dedicated areas or facilities [72,83]. Donkeys raised under temperate conditions are reported to need more protection in rainy and windy weather than horses, as the results of the adaptation of donkeys to semi-arid environments of Africa vs. continental climate, and Eurasian Steppe environments where horses evolved [86]. The grazed area must be close to the milking site [70,79] so that the proximity of pasture represents a constraint in the dairy equine enterprise and management of milking influences the feeding strategy. 

In intensive farming systems, the dairy mare and jenny are milked in ad hoc facilities equipped with sheep milking machines adapted to the equine mammary characteristics [15,68]. With trained animals and skilled operators, no difference was observed in the amount of milk harvested manually or mechanically per milking session, but milk microbial contamination can be reduced by the proper use of milking machine. This introduces a crucial aspect of the equine milk production and its commercialization, related to consumer safety.

### 3.2. Equine Milk: Hygiene and Health Issues

In Europe, equine milk is mainly commercialized at farm or by means of vending machines (raw milk), but it is also available at shops and supermarkets (pasteurized milk) or online (pasteurized and powdered milk) [22,71,87,88]. 

The risk associated with equine milk consumption is considered reasonably low when compared to bovine milk. The presence of pathogens, such as *Escherichia coli* O157, *Salmonella* spp., *Campylobacter* spp., *Yersinia enterocolitica*, *Brucella* spp., *Mycobacterium* spp., *Bacillus cereus*, *Cronobacter sakazakii*, *Streptococcus equi* subsp. *zooepidemicus*, *Rhodococcus equi*, *Streptococcus dysgalactiae* subsp. *equisimilis*, *Clostridium difficile,* and *Burkholderia mallei* is reported to be low [87]. However, the variable level of microbial contamination of equine raw milk, ranging from 3.0 to 5.87 log CFU mL^−1^ milk, warns against ineffective sanitization of equipment and facilities, as well as packaging and storing conditions of milk, even after thermal treatments [22,29,87]. 

For these reasons, while alternative processing for equine milk sanitation and shelf life extension are studied [22], thermal treatment is always recommended before consumption [87].

In regards to the mammary gland health status, the somatic cell count is reported to be below 50,000 cells mL^−1^ milk and mastitis is rarely observed in the dairy equine farm [29,87,89]. However, injuries or improper milking procedures reported for more intensive farming systems can affect the mammary health status [31,87].

Equine milk is gaining interest as an alternative food for sensitive consumers, so that high hygiene standards represents an important issue in the dairy equine enterprise, and it affects the labor costs for cleanliness of facilities, and areas frequented by the animals.

### 3.3. Feeding the Dairy Equine and Pasture Management

The nutritive value and the potential health-promoting properties of equine milk are related to the horse and donkey’s metabolic utilization of the diet. These monogastric species and hindgut fermenter herbivores are reported to be better utilizers of metabolizable dietary energy than ruminants, at high levels of cell wall [90]. It is also well known that the dietary influence on milk composition is more direct in the equine species than in ruminants [10,90]. Regardless of the farming system, as already mentioned, the common denominator in the diet is the presence of forages and pasture, whose management is crucial for dairy equine production and welfare [71,72,74,91,92]. Because of the evolutionary history of the two equine species, their different feeding behavior and metabolism should also be considered in relation to nutrient requirements, management of feeding, and their impact on land preservation.

#### 3.3.1. Feeding the Dairy Horse

According to Doreau and Martin-Rosset [10], no different approaches are required in feeding the dairy or nursing horse, as far as the energy and nitrogen requirements are concerned. The nutritional requirements of the lactating mare (600 kg body weight) are summarized in Table 3 [93]. To sustain the milk production, forages account on average for 50 to 80 percent of the dry matter of the diet, and they can supply 40 to 70 percent of the mare’s annual nutritional requirements [5,94]. The dry matter intake of mares (Table 3) depends on the quality of the diet at foaling [93]. At the onset of lactation, the voluntary intake of mares is reported to be high (20–30 g dry matter per kg body weight). However, the dry matter intake is scarcely a limiting factor for the mare to meet nutritional requirements [93].

The diet composition varies according to quality and availability of pasture and forages. In case of good grassland conditions, dairy mare foals generally in spring, just before they turn out, and use natural or sown pasture during the grazing season [95]. They are generally dried up in autumn (early October), after 190–210 days of lactation. During winter (110–120 days), the mares are fed a limited amount of hay of medium quality (organic matter digestibility, OMD = 50–55%) [93], and cereals, or a mixed diet based on straw, ad libitum, and hay of good quality (OMD = 55–60%). In case of harsh conditions, mare foals generally in early spring, one month before turning out. They graze pastures of uplands. They are dried off in autumn (late October), and grazed resources meet the requirements of animals over 9 months of lactation. In case of low productive areas, mares graze for about 60–70% of the total grazing season. In late autumn and early winter, mares graze refusals of cattle and sheep in the lowlands [93]. 

In the dairy horse enterprise, the strategy of the feeding system is based on pasture availability throughout the year and consists, generally, in matching the highest requirements of the animals with the maximum biomass production [96]. It must also include provisions of preserved feedstuffs to be used in case of particularly adverse climatic conditions. The main aim of the feeding strategy, notwithstanding the horse breed, is that dairy mares gain body weight in early lactation to nurse adequately the foal and to be rebred as soon as possible, to achieve a 12-month interval between two subsequent foalings [94]. Foals live with their mothers at pasture and they are allowed to nurse when mares are not milked. Table 4 shows the nutrient requirements of foals (600 kg of adult body weight) performing an optimal or moderate growth rate.

Mares should be managed at pasture with the aim to recover body weight and a proper body condition at drying off in late summer or fall, to ensure good nutritional conditions in pregnancy during winter [93]. Mares increase their body weight (+6–8%) during the last three months of pregnancy, as they are usually fed from 100% to 120% of their energy requirements, and during the first month after foaling (+3%) when they turn out in spring [93].

In extensive farming systems, grazing dairy mares should meet 80% of the total requirements over the 7-month lactation period. The animals use the vegetation regrowth from September until December. As already mentioned, in more intensive farming systems, hays or maize silage (30–35% dry matter content, 0.80–0.84 Horse Feed Unit per kg dry matter) and low concentrate supplementation are offered during winter. 

#### 3.3.2. Feeding the Dairy Donkey

Nutrient requirements and suggested allowances, nowadays available specifically for donkeys, are mainly devoted to working animals, i.e., used for transportation, small agricultural works, and equine therapy, and to animals at maintenance, i.e., companion animals, often castrated, hosted in international animal rescue charities, e.g., Donkey Sanctuary [72,79,97,98]. The available nutrient requirements of the dairy donkey are either based on results from one study on foal growth, or they are derived from domestic horse data, whose behavior and physiology are known to differ from those of the donkey. Consequently, they cannot be considered conclusive and need further investigation [20,72,79,98,99]. As reported by the US Research Council on equids, donkeys maximize their dry matter intake when good quality hay is offered [100]. For donkeys at maintenance, Raspa et al. [79] report a maximum dry matter intake per kg of body weight, declining from 32 to 12 g, with ad libitum diets based on either legume forages or barley straw. In order to prevent nutritional diseases, e.g., hyperlipemia and obesity, a diet high in fiber is suggested for companion donkeys at maintenance [99]. 

Because the mentioned lack of information in the specific literature on protein and energy requirements of lactating donkeys [20,72,79,98], the common strategy is represented by ad libitum administration of forage-based diets associated to a monthly evaluation of the body condition score [31,79]. 

In the dairy equine farming system, pasture should be always available for its positive effect on animal welfare and milk quality [79]; however, when jennies are milked, grazing time and quanti- qualitative availability of grazed areas are limited, as also observed for dairy mares [70]. According to preliminary results on the grazing behavior of Miranda breed jennies in mountain pastures, Couto et al. [101] observed that the activities in searching and prehension lasted, on average, 16 hours per day with a preferential intake of herbaceous species. However, up to 30% of the intake was represented by shrubs, probably because of a low grass availability. This suggests the interesting role of these autochthonous donkeys in preserving the pasture areas from degradation and fire risks [101]. It is also worth considering that grazing time does not significantly affect the daily dry matter intake of donkeys at maintenance, when they also have free access to preserved forages [102].

Results of a survey carried out on 12 dairy donkey farms in Italy confirm the inclusion of pasture in the lactating donkey diets always associated to hay administration [72], likely due to the limited availability of pasture. Cereals and/or mixed feeds, commercial or not, are also administered to lactating donkeys, and diets are frequently salt supplemented [31,72]. 

Other data from on field studies about milk production report a high feed intake (30–32 g dry matter per kg body weight) of dairy jennies at the first 3–4 months of lactation. Moreover, diets are characterized on average (on a dry matter basis) by a 70:30 forage-to-concentrate ratio, a protein content of 10–13 g per 100 g, and a digestible energy value of 8.5–10.0 MJ per kg [20]. 

After digestion, dietary fats, soluble carbohydrates, and proteins are mainly absorbed by the small intestine of equids. Due to the negligible biohydrogenation before absorption, the direct influence of the diet on the fatty acid composition of milk is expected. In this regard, the supplementation of the mares’ diet in late pregnancy, and early lactation with eicosapentaenoic acid (EPA) and/or docosahexaenoic acid (DHA) did not affect the linoleic and linolenic milk content, but it increased the arachidonic acid, EPA, and DHA milk concentrations [103]. However, in jennies, the transfer of n3 polyunsaturated fatty acids (PUFAs) from blood to milk is reported to be more efficient than that of n6 PUFAs [20]. For a nutritionally correct ratio of n3:n6 PUFA in equine milk, dietary lipid sources should be evaluated with regard to the fatty acid profile. Dietary factors can also influence the palatability of donkey milk. ‘Green’ aromatic notes and related compounds have been identified in milk when jennies were fed fresh forage [20].

The survey by Dai et al. [72] reported that the diet always includes hay (100% of farms) and pasture (about 92% farms) in non-lactating jennies. Concentrates and salt supplements are also administered, but in a lower percentage than during lactation [72]. Stallions, which are either grouped with females or housed individually, are mainly fed hay supplemented with mixed feeds, cereals, and salt or additives. Pasture availability is reported for about 70% of farms [72]. 

Nutrient requirements for foals are not defined and only rare data are available on growing rates in donkeys [104]. The administration of milk replacement formulas to foals is not common in the dairy donkey farm [72]. However, a highly digestible creep feed is usually distributed to nursing foals until one month of age, when dams are not milked [20]. Later, complementary feeds are administered to foals until weaning (7–12 months of age) [72]. Constant access to clean water and salt blocks is highly recommended for both foals and dams [20]. 

The welfare status of the animals needs to be constantly monitored by recommended indicators, such as body condition score, and hydration score [105]. Vaccinations and deworming are also recommended in all animals, along with regular hoof, dental, and health care treatments [105], even if they are not reported to be common practices in dairy donkey farms [72].

### 3.4. Pasture in the Dairy Equine Enterprise

Different systems of grazing management are possible: extensive vs. semi-extensive, associated or not with ruminants [106]. Depending on the grazing species and their nutrient requirements, the correspondence between animals and characteristics of the forage availability (in quality and quantity) is crucial for a sustainable use of the landscape [107]. As herbivore species, horses and donkeys have the ability to exploit large amounts of fibrous forages, often of low nutritive value in less favored areas, available for grazing and/or foraging. The ability of the equids to produce in high forage feeding systems is mainly explained by their distinctive features in selecting, consuming, and digesting forages and grazed resources [102], so that seasonal variations in grazing behavior and diet selection have been observed in mares [108]. In particular, equids show several adaptive abilities in harsh conditions when the total nutrient requirements can be achieved on a long-term period [102].

In free-ranging conditions, horses spend up to 70% of their time to consume available food resources and only 30% for other activities. This ingestive activity is usually distributed over several meals during the day and grazing also occurs during the night [70,109]. Moreover, grazing time can increase in autumn and in winter, and the length of grazing is in relation to the cell wall content of the sward [110].

In high forage systems, pasture is the major source of nutrients for dams and foals along the breeding cycle. Based on the type and composition of the grazing species, as well as on the carrying capacity of the pasture, the sustainable grazing period ranges from 100 to 130 days (Northern Europe), to 230 days (Central Europe) [5]. Especially in marginal areas, the most relevant management of horses and donkeys at pasture implies the evaluation of the nutritive value of forages available for the optimal animal response to match economic profits, technical feasibility, and ecological sustainability [107].

Pasture productivity varies according to the geographical zone and the climatic conditions. In Europe, the grazing period is usually limited in Northern countries by short summers. In countries of Central Europe, generally characterized by extensive grazing lands and high quality forages, long grazing periods are observed, while the grass growth is usually depressed by summer dryness in Southern Europe [5]. Table 5 shows the average chemical composition of pasture in selected areas of Europe. 

In addition, it should be noted that climate changes may affect the forage population dynamics, its nutritive value, as well as the growing and grazing seasons, so that different approaches in the management of land, animals, and forage resources may be required [112]. The forage intake depends on the quality of plant resources and their ingestibility, the time of grazing, the grazing activity, and the stocking rate, especially in multispecies herding situations. On this purpose, practical and flexible models have been studied for the assessment of a grazing pressure compatible with the conservation of pasture in less favored areas [113]. Moreover, the adoption of appropriate strategies is also recommended, such as rotational grazing, control of infesting species, safeguard from parasites diffusion in the sward, and fertilization (180 kg N ha^−1^) [107,108,114]. 

In extensive systems, characterized by low quality and poor productivity of natural pastures, the total requirements can be met using low grazing intensity, with a stocking rate of 0.3–0.7 animal ha^−1^, depending on the grass availability [106,114]. In more intensive systems of Central Europe, a concentrate supplementation is offered to horses, depending on their activity. Grass is plentiful until the beginning of July (beginning of the third vegetation cycle), then the production declines from mid-July to the end of August [94]. In Mediterranean regions, depending on the geographical area, the grazing season starts between April and May. A considerable reduction of the grass production is observed, depending on the variable rainfall in July, August, and early September. Then, up to the end of October, a regrowth of the grass can occur, offering the availability of fresh forages to foals in the weaning period [5].

**Figure 1:**
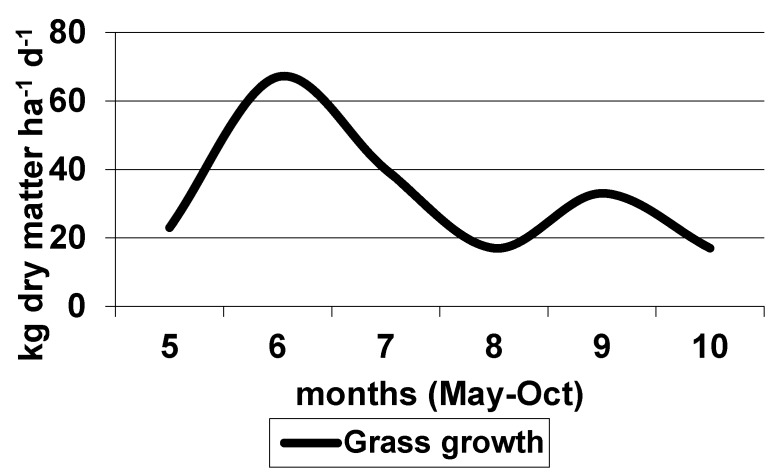
The average growth of pasture grass in Central Italy during the grazing period (modified from [94]).

**Figure 2:**
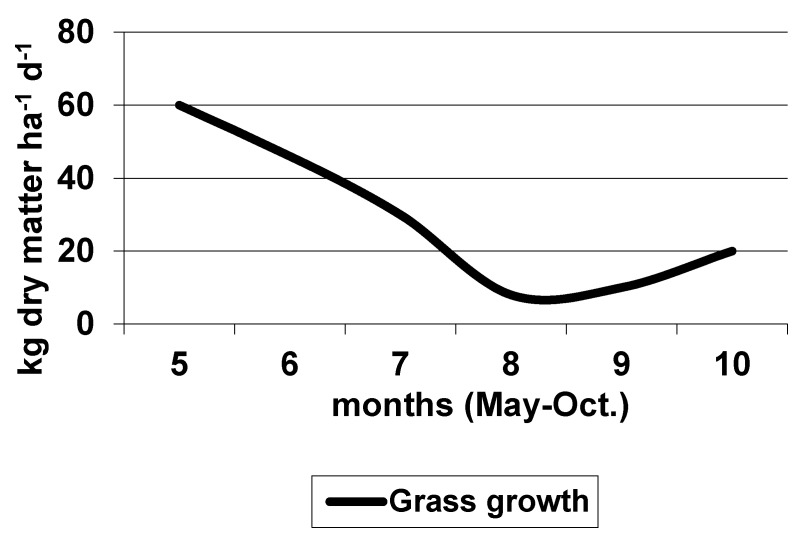
The average growth of pasture grass in Southern Italy (modified from [94]).

The average growth of pasture grass during the grazing period in Italy is shown in Figure 1 and Figure 2, respectively, for Central and Southern Italy pastures. It is interesting to note that in the pasture area located in Southern Italy, and in a mixed grazing system, including cattle, sheep goat, and horse, the sustainable stocking rate simulated in two subsequent years varied from 1.14 to 1.35 Adult Bovine Unit ha^−1^, due to different climatic conditions and carrying capacities of the pasture [107].

In order to achieve biodiversity and production goals in sustainable grazing systems of less favored areas, further management strategies include the reduction of the stocking rate, the periodic exclusion of the more degraded areas from grazing, the administration of complementary hay and concentrates, as well as the use of autochthonous breeds [115,116].

As a final consideration, the dairy equine enterprise, here described in its essential and promising traits, is the result of different environmental conditions, management strategies, and socio-economical aspects. Furthermore, no data on the evaluation of the economic impact of the dairy equine milk production are available in literature. However, besides labor, feeding, housing, and milking facilities, the evaluation of costs should also include those related to availability of infrastructures on pastures and marginal areas, and social costs of labor and bureaucracy, whose incidence can be relevant and different among countries [92]. Moreover, among the immaterial benefits, the impact of the dairy equine enterprise to environmental issues, such as landscape safeguard and biodiversity preservation, should also be included in a costs-to-benefits ratio evaluation [117], as also reported for horses used for tourism and work [3]. Alternatively, a price premium, based on environmental standards and labels, should be recognized to the products of the dairy equine enterprise.

## 4. Conclusions

The dairy enterprise involving equids, here discussed in its essential traits, represents a promising activity for the micro-economies of marginal areas around the world, because of its potentialities in human nutrition, biodiversity, and landscape preservation. Notwithstanding the advances of knowledge on milk nutritional and safety characteristics, as well as the improvement of technical skills in milk management, in depth studies are still required, especially in terms of animal nutrition and feeding. A better understanding on nutrient requirements of the dairy equid at pasture in heterogeneous and marginal areas will boost the interest toward endangered equine breeds, their milk, and their habitat. Positive relapses would in fact include the protection of plant diversity in the achievement of a productive and sustainable use of the landscape. Among the innovations for sustainable agriculture, the production of equine milk and derivatives with high nutritional value and health promoting properties should be therefore considered a promising extension of the equine industry for the modern and future society.

## Figures and Tables

**Table 1:** Average milk gross composition and energy content from different species ^1^.

Item	Horse	Donkey	Human	Cow
Total solids, g kg^−1^	103.1	95.3	125	127
Fat, g kg^−1^	10.3	7	35	41
Protein, g kg^−1^	16.8	16	12	34
Lactose, g kg^−1^	63	66	64	48
Ash, g kg^−1^	4.2	4.1	1.9	7
Gross energy, MJ kg^−1^	1.98	1.75	2.69	3.19

^1^ Sources: [15,26,28,29,30,31].

**Table 2:** Daily milk yield (kg/d) reported in literature for horse and donkey from d30 to d180 of lactation ^1^.

Item	Horse	Donkey
Mean value	11.66	2.68
s.d.^2^	5.3	1.96
Min	3.9	0.72
Max	17.2	6

^1^ Sources: Horse: [15,80,82,83], Donkey: [23,25,30,31,72,76,77,78,79,80,84]; ^2^ standard deviation.

**Table 3:** Recommended nutrient requirements and intake for lactating mares (600 kg body weight) [93].

Lactation, Month	Milk Yield, kg d^−1^	Horse Feed Units *, n d^−1^	Horse Digestible Crude Protein **, g d^−1^	Dry Matter Intake, kg d^−1^
1st	18	10.1	1131	13.5–18.0
2nd	19.8	10.3	1091	15.0–19.0
3rd	19.2	9.6	1030	15.0–19.0
4th	17.4	9.1	844	13.5–18.0
5th	13.2	7.9	629	12.5–15.0
6th	12	7.6	603	10.5–13.0

* Horse Feed Units (UFC) = 9.42 MJ Net Energy; ** Horse Digestible Crude Protein (MADC).

**Table 4:** Recommended nutrient requirements and intake for foals (600 kg adult body weight) at 3–6 months of age with a growth rate optimal or moderate [93].

Body Weight, kg	Gain, g d^−1^	Horse Feed Units *, n d^−1^	Horse Digestible Crude Protein **, g d^−1^	Dry Matter Intake, kg d^−1^
249	1000–1200	6	647	6.0–8.0
207	800–900	4.8	497	5.5–7.5

* Horse Feed Units (UFC) = 9.42 MJ Net Energy; ** Horse Digestible Crude Protein (MADC).

**Table 5:** Chemical components and estimated energy content of pasture in European areas. Values expressed on a dry matter basis [5,111].

Country	Crude Protein, g kg^−1^	Crude Fiber, g kg^−1^	Horse Feed Units *, n kg^−1^
Finland	200–230	180–200	0.69–0.73
France, lowlands	131–168	244–276	0.76–0.82
France, uplands	111–166	223–304	0.66–0.92
Italy, lowlands	85–159	242–325	0.67–0.90
Italy, uplands	117–155	285–345	0.63–0.85

* Horse Feed Units (UFC) = 9.42 MJ Net Energy.
